# Experiences of being a manager in the municipal sector in rural northern Sweden

**DOI:** 10.1080/17482631.2022.2139346

**Published:** 2022-10-25

**Authors:** Sofia Asplund, Johan Åhlin, Sture Åström, Britt-Marie Lindgren

**Affiliations:** Department of Nursing, Umeå University, Umeå, Sweden

**Keywords:** Experiences, managers, municipal sector, qualitative content analysis, rural area

## Abstract

**Purpose:**

The purpose of this study was to describe experiences of being a manager in the municipal sector in rural northern Sweden.

**Methods:**

Semi-structured interviews were performed with 15 managers working in the municipal sector. The interviews were subjected to inductive qualitative content analysis.

**Results:**

The managers’ experiences were interpreted in the main theme, *Striving for stability on shaky ground*. They struggled within slimmed-down organizations with substitute shortages and reduced means. They expressed being burdened by conflicting demands, a work overload, work conflicts and a requirement to be present. They had to endure the negative consequences of working in small municipalities, and handling health and family life impairments. The managers expressed the importance of being strengthened by inner and outer resources, such as being motivated, having social support and finding strategies to cope. They also expressed benefits of working in small municipalities, such as great opportunities to influence and efficiency in decision-making.

**Conclusion:**

This study illuminates managers’ adverse psychosocial working conditions, insights into working in a small municipality in the rural context and also the importance of organizational support. Future studies could focus on applying adapted workplace support interventions among managers in the municipal sector in rural northern Sweden.

## Introduction

The Swedish municipal sector employs almost 20% of the employees in the labour market, where school, preschool and elderly care are the dominating areas. Sweden’s 290 municipalities have their own self-governing local authorities with a high degree of autonomy, including the right to determine the taxation rate among their citizens. The municipalities have an elected assembly, the municipal council, which takes decisions on municipal matters. Every municipality, regardless of size, is bound by law and regulations to offer a number of public services, including childcare, schools and elderly care (Swedish Association of Local Authorities and Regions, 2022). The municipal sector has the highest rate of long-term sick leave compared to other sectors, and women have a sick-leave rate twice as high as that of men. The sick leave rate is highest in elderly care, followed by preschool, and municipalities in rural northern Sweden have the highest sick-leave rate in the country (Swedish Association of Local Authorities and Regions, 2021). Many of the Swedish municipalities have been facing a population decline for several years. Shrinking municipalities are a concern primarily for rural municipalities, often small in population but large in land area, and often located far away from the growth regions. Young and highly educated people move from the rural municipalities, leaving an older and lower educated population. The financial and organizational consequences of depopulation are described as troublesome; centralizing the care of the elderly, making extensive budget cuts and closing schools are some of the consequences reported. Rural municipalities in northern Sweden have described shortages in the workforce due to depopulation, especially in certain sectors, and difficulties recruiting, for example, certified teachers and nurses (Nyström, 2021; Syssner, 2020).

Managers have an important role in the organization, and the leadership of managers is important for the work environment, work performance and occupational health for all employees (Leithwood et al., 2020; Swedish Agency for Work Environment Expertise, 2020). A manager needs to have in-depth knowledge of laws and regulations regarding the organizational and psychosocial work environment, which determine the manager’s legal responsibilities and influence how the manager has to organize the work. The manager should also have knowledge of how to prevent and manage unhealthy workloads (AFS 2015: 4, p. 4). Research has shown that managers often try to balance various interests in their daily work. An organizational and psychosocial work environment with an excessive workload and little influence, and a negative balance between work and family life, can lead to work-related stress among managers. Lower-level managers have often less influence over their work than higher-level managers (Løkke & Madsen, 2014), and lower-level managers report more work-related stress and higher risk of exhaustion and sick leave compared to higher-level managers. Exhaustion is more common among female managers than male, indicating that the total workload is heavier for women, who often take a greater responsibility for family life (Björklund et al., 2013). Work-related stress among managers has also been negatively associated with work-related stress and ill health among employees (Skakon et al., 2010). It seems important for the employer to pay attention to the managers’ working conditions in rural northern Sweden and to signs of work-related stress, in order to promote occupational well-being.

The job demands–resources model (JD-R model; Demerouti et al., 2001) is widely used to examine predictors of employee well-being, engagement, and individual and organizational outcomes. The JD-R model serves as a useful framework for categorizing and exploring predictors of work-related stress. Job demands have been associated with burnout, negative organizational outcomes and poor health. In contrast, job resources have been associated with work engagement, positive organizational outcomes and increased well-being (Bakker et al., 2014). High job demands, workplace violence and low work time are some of the most important factors for risk of stress-related exhaustion and long-term sick leave. Working in human service organizations involves a higher risk of sick leave than working in other organizations (Aronsson et al., 2017; Seidler et al., 2014). In recent years, there has been a large increase of long-term sick leave due to mental ill health among managers in Sweden (Previa, 2019), and the sick-leave rate is higher among managers in the municipal sector compared to other sectors (Andrén, 2018). Managers in the municipal sector have large areas of responsibility, including managing staff, budget, local level decisions in the operation, and are accountable for the work environment of the employees. The managers are divided into three levels: senior manager, middle manager and first-line manager, where the majority are first-line managers (71% women). First-line managers are responsible for day-to-day operations at workplace levels. (Swedish Association of Local Authorities and Regions, 2021). Unequal working conditions have been described among managers in the municipal sector, where managers in feminized care service work have fewer resources and less support, and a higher number of subordinates, compared to those in masculine municipal services such as the technical sector (Björk & Härenstam, 2016). Over time, there has been a negative development of job demands, decision-making authority and social support among managers in the female-dominated sectors such as education, health and social care in Sweden (Cerdas et al., 2019). Swedish managers working in school and elderly care sectors have described an imbalance between demands and resources, with a negative impact on health, the service they deliver, goal fulfilment, motivation and their willingness to stay in the profession. The number of subordinates a manager is responsible for is of great importance for the manager’s work environment. It is common that managers in preschool, school and elderly care are responsible for over 35 subordinates, associated with an increased workload, and the number is twice as many compared to the technical sector (Corin & Björk, 2017).

In summary, the leadership of managers is important for the work environment, work performance and occupational health for the employees (Swedish Agency for Work Environment Expertise, 2020). Research has shown that there are adverse psychosocial working conditions in Swedish municipal organizations. Managers in the municipal sector are exposed to an organizational and psychosocial work environment with high demands and inadequate resources, including risk of work-related stress and sick leave, for both managers and employees (Björk & Härenstam, 2016; Swedish Association of Local Authorities and Regions, 2021). Rural areas in Sweden are facing challenges when it comes to decreased economy, depopulation and difficulties in recruiting staff (Syssner, 2020). Few qualitative studies in rural northern Sweden have focused on managers’ perspectives, and such a study can contribute to an increased understanding and important knowledge in the development of methods aiming to improve managers’ occupational well-being and work performance, and organizational productivity.

### Aim

The aim of this study was to describe experiences of being a manager in the municipal sector in rural northern Sweden.

## Methods

We used a qualitative design and collected data through individual semi-structured interviews in order to describe experiences of being a manager in the municipal sector, which we analysed using qualitative content analysis (Graneheim et al., 2017; Graneheim & Lundman, 2004; Lindgren et al., 2020).

### Context

This study is part of a larger study, conducted in collaboration with municipal authorities in two rural municipalities in northern Sweden, where quantitative data have earlier been described (Asplund et al., 2020). The two municipalities were characterized by high rates of high long-term sick leave among the employees, due to stress-related disorders. One municipality has about 3100 inhabitants in an area of 1600 square kilometres (~618 square miles), and the other has about 12,200 inhabitants in 5500 square kilometres (~2125 square miles; SCB, 2021). One of the municipalities has experienced one of the highest population decreases in the country (almost 30%) and reduction in school pupils of 45% in the years 1975–2018 (Syssner, 2020).

### Data collection

The inclusion criteria included working as a manager in the municipal sector, and not being on sick leave. The eligible participants comprised in total 30 managers, 18 women and 12 men. A list of the managers’ email addresses was provided by the municipal administration. An information letter was sent to the possible participants by email, followed up with a personal call about a week later. A sample of 15 managers consented to participate in the study: 9 women and 6 men, aged between 35 and 62 years (median = 46). They served at different levels, where the majority were principals at schools and preschools and managers within nursing homes. The managers’ total length of experience varied between less than a year to over 28 years (median 6.5).

Individual semi-structured interviews (Brinkmann & Kvale, 2015) were carried out between February and April 2019, at a location of their choice, either at their workplace or at the town hall. The interviews lasted between 42 and 70 minutes (median 54). The first author performed the interviews using an interview guide with four open-ended questions: “Please can you tell me about your experiences of being a manager”, “Please tell me about your work conditions”, “Please tell me about managing the organizational and psychosocial work environment”, “Can you tell me about your experiences of work-related stress?” Probing questions such as “Can you tell me more about that?” and “In what way?” contributed to richer descriptions and deeper understandings of the interviewees’ experiences. The interviews were digitally recorded, and a secretary skilled in transcribing interviews transcribed them verbatim. The length of the transcriptions were 15–26 pages (median 19).

### Data analysis

Inductive qualitative content analysis was used to interpret the textual and underlying content of the interview data (Graneheim et al., 2017; Graneheim & Lundman, 2004; Lindgren et al., 2020). The interviews were read through several times to get an overall view of the data. The texts were transferred to the MAXQDA software programme for qualitative data analysis. The first author divided the text into meaning units consisting of individual words, sentences or short paragraphs relevant to the aim; condensed the meaning units to preserve their core meanings and manifest content; and labelled them with codes describing their content.

The codes were sorted by content, abstracted and interpreted into ten subthemes, four themes and one main theme. For example, codes such as “high number of work tasks”, “lack of time for lunch”, “unreasonable expectations from the management”, and “feeling insufficient” were grouped under the subtheme *facing a work overload* and included with other subthemes under the theme of *Burdened by conflicting demands*. Together with three other themes we interpreted these under the main theme, *Striving for stability on shaky ground*. All authors discussed the analysis until we reached agreement.

### Ethical considerations

This study was approved by the Regional Ethical Review Board, Dnr 2017/495-31. We followed the 2013 Declaration of Helsinki recommendations to maintain human rights and adhere to scientific principles throughout the study. The participants were informed, both verbally and in writing, that their participation was voluntary, and that they could withdraw from the study at any time without giving any reason. The participants were informed about the General Data Protection Regulation (GDPR) principles. They were also informed that their data would be treated confidentially by the researchers, by coding the interviews and establish a code list, and that none from the municipal organizations would get access. Signed informed consent forms were provided by all participants. The researchers were working at a university located in another municipality, and had no relation to the participants in the study.

## Findings

The findings from the analysis and interpretation of the participant’s experiences of being a manager revealed one main theme: *Striving for stability on shaky ground*. The findings also revealed four themes: *Struggling within a slimmed-down organization, Burdened by conflicting demands, Endure the consequences*, and *Strengthened by inner and outer resources*. Each theme consisted of two or three subthemes, as presented in Table I.

**Table I.** Overview of the subthemes, themes and main theme revealed in the analysis.

Subthemes	Themes	Main theme
Facing a substitute shortage	Struggling within a slimmed-down organization	Striving for stability on shaky ground
Managing reduced means		
Facing a work overload	Burdened by conflicting demansds	
Dealing with workplace conflicts		
Feeling required to be a present manager		
Feeling trapped and alone in the small municipality	Endure the consequences	
Facing impaired health and family life		
Feeling motivated and supported	Strengthened by inner and outer resources	
Seeing the benefits of the small municipality		
Finding strategies to cope		

### Striving for stability on shaky ground

The participants described *striving for stability on shaky ground*. They experienced struggling within a slimmed-down organization with inadequate resources, a substitute shortage and reduced means. They also experienced being burdened by conflicting demands, a work overload and work conflicts, and felt required to be a present manager. Being a manager in the municipal sector meant having to endure the consequences, where they felt trapped and alone in the small municipality, as well as facing impaired health and family life. The participants also expressed positive experiences, where they were strengthened by inner and outer resources as they were feeling motivated and supported, seeing the benefits of the small municipality and striving to find strategies to cope.

#### Struggling within a slimmed-down organization

The participants described *struggling within a slimmed organization* when working in the municipal sector, which meant facing a substitute shortage as well as managing reduced means.

##### Facing a substitute shortage

The participants described facing a substitute shortage, which had major consequences for the working environment. A large part of the working time was spent on calling substitutes and changing the employee work schedule, to make sure that there was enough staff. The substitute shortage was described as a source to stress among the managers. When their colleagues were on vacation, the managers were also struggling to cover their colleagues’ worker shortages as well as their own. One participant said,

The substitute situation is chaotic, and when we do not get hold of people, it can take an incredible amount of time for a manager to call out, redirect schedules and find solutions. (IP2)

The lack of substitutes forced the managers to hire substitutes without education and experience, as well as those with poor language skills. This forced the regular staff to supervise untrained substitutes at all times. Some managers employed an extra staff person who could work where there was a staff shortage. It was described as “better to overstaff” for a better working environment. Some participants reported that the inadequate resources resulted in having to fill in as substitutes themselves for the employees. There were also participants who described having a better situation with substitutes, and having employees who handled calling substitutes themselves.

##### Managing reduced means

The participants described managing reduced means, and could not affect their budgets, which has decreased more and more each year. If improvements were made at one workplace, they struggled with reduced means for other workplaces, which led to deteriorations there. Although managers were expected to perform well, inadequate resources affected the work environment negatively. One participant said,

Now we will get further budget cuts, which affects everyone in the workplace. How can you be expected to succeed with the managerial work? (IP2)

The reduced means also affected the opportunity for education; some participants avoided asking their supervisors for approval to attend courses, since they knew that they would be refused. The participants described being dissatisfied with both salary and status, and felt that the organization did not invest much in the managers. The economic situation in the municipalities was tough; the elderly care has declined for several years, leading to worry that the school sector would likewise be impoverished. Support functions, such as administrative assistants had been removed. The workplace wellness hour, where the employees are allowed to set aside one hour of work time a week for physical activity or other health promotive activities, had also been removed in one municipality. It was described that the high sick-leave rate was an effect of reduced means and a sign of ill health among the employees. The participants struggled in an organization where the municipal management was perceived as weak and unclear, which affected the managers in a negative way. Progress in the municipal sector was slow. One participant said,

Being a manager in the municipal sector means working on an uphill slope. (IP1)

#### Burdened by conflicting demands

The participants felt *burdened by conflicting demands*, when various demands exceeded their ability to cope within the time available, described as facing a work overload, dealing with working conflicts and feeling required to be a present manager.

##### Facing a work overload

The participants related that they experienced facing a work overload, where work tasks and responsibilities exceeded the time available. They were even assigned work tasks outside their responsibilities and were expected to be a caretaker, an expert and an economist. Being a manager was described as “not easy”. The participants were often responsible for several workplaces and a high number of subordinates. They described that the work overload meant that there was too little time to be able to perform their work in the way they wanted to, something that was hard to face. They did not have time to take lunch, or time for intended educations during working hours. They were burdened by a bad conscience when delegating work tasks to others, and some felt insufficient as a manager. They also described feeling burdened by high demands from superiors as well as from the employees, who expected the manager to do everything. The participants described it as difficult to live up to what the politics demanded, which in some cases involved implementation of new things all the time. The organization was constantly changing, and it was difficult to see any development. The participants described it as demanding having to cooperate with different authorities. The work overload also involved IT demands, with a constant flow of emails and phone calls, even in the evenings and weekends. One participant said,

Sometimes it feels like the managers are busy keeping their head above water. There is no time for looking ahead. (IP10)

The participants experienced demands to have good knowledge about laws and provisions regarding the organizational and the psychosocial work environment. They were responsible for working systematically with the organizational and psychosocial work environment, but there was often a lack of time for proper documentation. There were also challenges with poor physical work environments, for example, inadequate premises in schools and nursing homes, especially when renovations were taking place.

##### Dealing with workplace conflicts

The participants described feeling burdened by dealing with workplace conflicts, which were difficult to handle, causing stress and frustration. Dealing with conflicts was part of working as a manager, something a manager cannot avoid or be afraid of. However, it was also unclear what the managers and employees could expect from each other, which created conflicts. In times of organizational changes, the managers often experienced working with a lot of resistance from the employees, which led to dealing with conflicts, experienced as difficult. The managers were greatly affected by conflicts within the staff group, where they often had to deal with replacing employees and moving them to other sections to solve conflicts. Employees got angry and cried sometimes when the managers made organizational changes. The participants described it as important to deal with problems within the employee work group. Otherwise, negative attitudes of employees could infect others, resulting in everyone being in a bad mood. One participant described,

I am shocked at how the employees talk to each other, and also to the first-line manager. Everyone is angry at each other, I can understand that it is leading to employees being on sick leave. (IP1)

Working as a manager also involved being burdened by disciplinary actions such as dismissals, often experienced as hard to deal with. Some managers were involved when there were conflicts between teachers and students, and also had to deal with demanding relations with parents and relatives.

##### Feeling required to be a present manager

The participants described that they experienced feeling required to be a present manager, important for the safety and well-being of the employees. Just taking a coffee with the employees and asking how they were doing could solve small concerns before they became large. Also, to be able to work with organizational development, the managers described it necessary to be present. Some participants stated that their supervisors had demanded that they spend at least 20%–25% of their work time among the employees, which could be difficult to achieve, especially since there could be a geographical distance between the units. Managers (often first-line managers) responsible for over 50 employees often described it as difficult being a present manager. One participant said,

It is stressful being responsible for many employees. With fewer employees, I could be a more present manager. (IP15)

Being a present manager was also required to observe signs of stress among the employees and to try to handle this before it resulted in sick leave. All employees were needed in the organization, so there was big problem when employees were on sick leave due to stress.

#### Endure the consequences

The participants described having to *endure the consequences* of their work situation, meaning feeling alone and trapped in the small municipality, as well as facing impaired health and family life.

##### Feeling trapped and alone in the small municipality

The participants described feeling trapped and alone in the small municipality, with few opportunities to develop and grow, or to find other managerial jobs. It was also hard to move to the small municipality from another place, meeting resistance when trying to make a career. One participant said:

Sometimes it feels like it does not matter how well you perform at work as a manager in this small municipality. You have to endure that there is a predetermined order to make career, and there are always several managers in line waiting before you. (IP3)

There were also difficulties for newcomers moving to the municipality, since they did not feel welcomed in the community, and as a consequence, moved away after a while. It was also expressed that there often were few applicants for managerial positions, and that the organization sometimes had to settle for managers without experience or proper education. They also described that it was difficult knowing who was reliable in the small municipality, since everyone knew everyone, and what was said in one place came out elsewhere. Their employees were also related to each other, they were friends with co-workers and managers on Facebook and the close relationships could complicate the managerial work. The managers expressed that many employees avoided talking about private problems; they were afraid that these could out in the small municipality, and they could therefore refuse support from the occupational healthcare.

The participants described feeling alone in their position with lack of support from co-workers and managers, as well as from other support functions. Some reported thinking of looking for a new job, since their work situation with lack of support was unsustainable to endure. They also described feeling alone in stressful situations, when crises appeared in their workplace or when being exposed to threats of violence. One participant said,

My manager does not have time to give any support, I have to solve most things on my own. In this small municipality, there are not enough support functions for managers. (IP3)

The participants described a wish for more help from different support functions, such as administrators, the HR department and the occupational healthcare. One municipality was missing an HR specialist, and in the other municipality the HR department was very small, and as a consequence, the managers felt alone in their positions. The managers could not access the occupational healthcare as they needed; all orders for occupational health had to be approved by the HR department, making the managers feel trapped.

##### Facing impaired health and family life

The participants described facing impaired health and family life as consequences of their work situation. They reported experiencing poorer health since becoming a manager. The stress at work led to less energy or time for physical activity in their free time, poorer diet and weight gain. Some managers had not been able to use the wellness hour in several years. One participant said,

I would just be stressed to use the wellness hour, and be thinking of all the work I have to finish. (IP13)

The participants described an impaired family life, not having enough time or energy to do things together with their families, and struggling with a bad conscience. It took several weeks to regain energy during their holidays, making their family life affected. The participants described never leaving work completely and being stressed in their free time over things not done at work. Some managers described feeling sad when going home from work and endured being in the middle of work and family demands.

#### Strengthened by inner and outer resources

The participants described *being strengthened by inner and outer resources*, which they described as feeling motivated and supported, seeing the benefits of the small municipality, and finding strategies to cope.

##### Feeling motivated and supported

The participants described feeling motivated and supported at work. They described work as a health factor and felt content with the managerial work. Being a manager could be stressful, but despite that, they felt an inner motivation and happiness at work. One participant said,

I really love my work, I feel satisfied working as a manager in the municipal sector. (IP8)

The participants described learning something new every day, which was a positive experience. They liked having lots of projects on the go, and felt strengthened by feeling confident in their role as a manager. They would not want to change jobs, even though there was a heavy workload. It was also described as important to be satisfied with the work performance and to be optimistic about the future.

The participants described the importance of feeling supported by managers and co-workers. One manager said,

I often say that I would not survive without my co-workers. We work very closely, talk about situations and problems, give support and help each other. It feels very good to have support. (IP4)

Older managers were described as role models for younger managers. The managers could also ask the employees for advice, which was highly appreciated. Other important resources for support were the occupational healthcare and HR, often giving support to managers and individual employees, helping in time of conflict, sick leave and the rehab process. Other received support came from manager coaches, psychologists and projects aiming at increasing support for managers.

##### Seeing the benefits of the small municipality

The participants described seeing the benefits of the small municipality. There was good cooperation in the organization, and also great opportunities to influence, and the managers felt strengthened by being allowed to create their own solutions and make their own decisions. Work decisions were described as efficient in a small municipality; the managers could sit down with the employees and make decisions immediately. It was expressed that the small municipality had a breadth of knowledge that larger municipalities did not have, since they were more specialized. One participant said,

Working as a manager in a small municipality is like being a general practitioner. We have a broad knowledge and skills that a large municipality does not have. (IP 10)

##### Finding strategies to cope

The participants described finding strategies to cope with their work situation. They described it being important to distance themselves from the employees and the workplace at times to cope, by closing the office door, turning off the mobile phone, working from home one day a week or having an office at another location. The managers also warned the employees when there would be stressful times. It was also described as important to prioritize work and to delegate work tasks to the employees. They also expressed the importance of stopping some development work, since it was too hard to handle everything new. One participant said,

A manager never finishes the job; I can work myself to death. Therefore, I have to set boundaries and decide what is a reasonable workload. (IP10)

The participants also had to let go of negative energy and not take criticism so personally; otherwise, they would be at risk of ill health and sick leave. They chose to not interfere in employees’ private life problems, and to not blame themselves or the workplace when employees took sick leave.

## Discussion

This study described experiences of being a manager in the municipal sector in rural northern Sweden. The main theme, *Striving for stability on shaky ground*, summarizes our overall interpretation of the managers’ experiences of struggling in a slimmed-down organization where inadequate resources and conflicting demands had a negative spillover onto health and family life. Even though the ground was shaky, they found stability when being motivated and supported at work, as well as when finding coping strategies. They expressed how working in the small municipality gave rise to disadvantages, but they could also see and appreciate the advantages.

The managers in this study described slimmed-down organizations with inadequate resources, where a lack of substitutes and means caused, for example, difficulties in performing well at work along with work-related stress. Our findings are in accordance with the JD-R model, in which a lack of job resources cannot buffer the effect on high job demands, which can lead to difficulties in coping and reaching work goals, as well as health complaints and exhaustion (Bakker & Demerouti, 2017). Lack of resources has also been described in previous research among managers in the municipal sector (Corin & Björk, 2016; Elomaa et al., 2021; Jonasson et al., 2019; Landstad & Vinberg, 2013). Inadequate resources have been described as leading to difficulties in influencing the managers’ own work situation (Landstad & Vinberg, 2013), work-related stress (Elomaa et al., 2021), lack of competence (Corin & Björk, 2016) and manager turnover (Corin et al., 2016), as well as difficulties in providing good quality of care for the elderly (Jonasson et al., 2019). This indicates the importance of having enough resources to meet the demands to prevent the negative consequences of an unhealthy work environment for managers working in the municipal sector in rural northern Sweden.

This study found that the managers felt burdened by conflicting demands, a work overload and workplace conflicts, and they felt required to be a present manager. Conflicting demands have been described in other research among managers in the municipal sector (Ekholm, 2012; Elomaa et al., 2021; Jonasson et al., 2019; Landstad & Vinberg, 2013). In rural areas, managers working in school and health care units witness conflicting demands from senior officials, politicians, service users and relatives. A high workload, including being responsible for a high number of subordinates, made it difficult to be a present manager, especially since the units could be scattered across widespread geographic areas (Landstad & Vinberg, 2013). Similar challenges with geographical distance and a high number of subordinates were also described in our study, as well as ethical challenges when it was difficult to live up to the conflicting demands. Other research has described ethical conflicts at work among middle managers, with contradicting demands delivered by senior managers and politicians, healthcare professionals, and the elderly and their relatives, as well as control by laws and guidelines (Jonasson et al., 2019). The managers in the present study described feeling unable to be a present manager due to a heavy workload and increased administrative tasks. Similar findings have been described in previous research, and when the managerial role expanded, more time was spent on administrative tasks and documentation, resulting in less time to be a present manager (Corin & Björk, 2016; Kristiansen et al., 2016). Difficulties in living up to various demands and expectations (Ekholm, 2012), as well as a high workload and interpersonal conflicts (Elomaa et al., 2021), have been described as causes of work-related stress, and high demands and low control have been associated with a five times higher risk for emotional exhaustion (Jonsdottir et al., 2020). This is in line with the present study, which also highlights the high workload among managers, including that they were burdened by workplace conflicts. This is also in accordance with the JD-R model, where high job demands can lead to poor organizational outcomes and exhaustion (Bakker & Demerouti, 2017). In order to promote occupational health among managers in the municipal sector in rural northern Sweden, it seems important that the politicians and the municipal management focus on offering managers authority and support to deal with conflicting demands, workplace conflicts, workload and the possibility to be a present manager.

In the present study, the managers endured the consequences, felt trapped and alone in the small municipality and faced an impaired health and family life. Previous research has found that managers working in the municipal sector in rural Sweden felt alone and lacked support, as well as experiencing health problems as signs of stress-related illness. It was difficult finding time and energy for physical activity, both at work and in their free time (Landstad & Vinberg, 2013). Another study among principals in rural Sweden showed challenges when everyone knows everyone in the municipality. The principals knew many people personally, for example, politicians and parents, which led to burdensome expectations on the principals. They felt pressure from the surrounding community and felt that they let people down if they looked for another job (Nordholm et al., 2022). This in line with the present study where the managers described the disadvantages of the small municipality—when everyone knew everyone, it complicated the managerial work—and also lacked support from co-workers and managers as well as from other support functions. Lack of support has in other research been associated with boundary-less assignments, overtime work and risk of illness (Corin & Björk, 2016), as well as the decision to leave a job (Corin et al., 2016; Cregård & Corin, 2019). It seems important that the municipal management and politicians gain knowledge and awareness of managers’ exposed positions and offer them support and supervision in their role as managers in rural areas.

The managers in this study were strengthened by inner and outer resources, such as motivation, social support and the benefits of the small municipality, as well as coping strategies. According to the JD-R model, job resources are especially important for influencing motivation when job demands are high, and motivation has a positive impact on job performance and preventing exhaustion (Bakker & Demerouti, 2017). Our findings seem to be in accordance with the JD-R model, where motivation was described as important among the managers in order to deal with a stressful job, to thrive and to stay in their municipal employment. Similar findings in our study have also been reported in other research among managers in municipal organizations in Sweden, where the managers described being motivated and enjoying their work (Albinsson & Arnesson, 2018), as well as being proud and finding their jobs meaningful (Hagerman et al., 2019). Our study found that support from supervisors, co-workers and other support functions were described as important among the managers to strive for stability in their work situation. Previous research among public service managers in Sweden found that the supported and motivated managers performed better at work, with better health (Berntson et al., 2012). Other research has also described the importance of support to managers (Corin & Björk, 2016; Elomaa et al., 2021; Jonsdottir et al., 2020; Persson et al., 2021), to deal with the demanding job as a manager and to solve problems (Corin & Björk, 2016). Support has been described as buffering job strain and enhancing well-being (Jonsdottir et al., 2020), and also as helping to cope with work-related stress (Persson et al., 2021). Our study described different coping strategies among the managers, for example, prioritizing work and setting boundaries between work and private life. This is in line with other research, describing the importance of coping strategies for Finnish principals*’* occupational well-being, for example, setting boundaries at work, keeping a positive attitude, keeping work and free time in balance, physical activity and spending time with family (Elomaa et al., 2021). Further, findings in the current study showed that the managers were dealing with stressors, such as problems among the employees and personal criticism, by avoidance. Avoidance coping has been described in previous research as a strategy whereby the manager escapes stressors, described among Swedish managers in dealing, for example, with workplace conflicts and bullying (Björklund et al., 2019) and work-related stress, and that the strategy to distance themselves could be a sign of lack of support from supervisors (Skagert et al., 2008). This indicates the importance of support, motivation and coping strategies for managers working in the municipal sector in rural areas in order to enhance occupational well-being and work performance.

The results from this study indicate that a large part of the responsibility seems to be placed on the managers themselves to handle. Research on managers in the municipal sector has described the importance of shifting focus from individual to organizational level in order to create conditions for the managers to be successful at work. A supportive organization, that enables a present leadership by having a reasonable number of subordinates, and also enables a systematic work environment management are some important factors (Corin & Björk, 2017). The results from this study seem to indicate the need to improve the work conditions for managers in municipal organizations in rural northern Sweden, possibly by workplace-oriented interventions. The importance of support in organization-level interventions to improve working conditions, well-being and performance has been described among Swedish public managers (Härenstam et al., 2022). A longitudinal study of workplace-oriented interventions on individual and organizational level among managers and employees working in municipal home care, elderly care and schools showed a limited effect on health and psychosocial working conditions (Vinberg et al., 2015). Several barriers, such as high workload, lack of senior management support, politically initiated projects and organizational changes, have been described when implementing a health-promoting leadership programme in municipal organizations in Sweden (Larsson et al., 2015). Despite these discouraging results from previous interventions, we believe that support from politicians and the municipal management is of great importance for managers, along with providing managers with tools to strengthen their role in the municipal organizations and to feel prepared when striving for stability on shaky ground.

### Methodological discussion

To achieve trustworthiness in qualitative research, we reflected on credibility, dependability and transferability (Graneheim et al., 2017; Graneheim & Lundman, 2004). To enhance the credibility, as well as the data and analysis matching the intended aim, we included participants who best represent or have knowledge of the research topic. We also made sure to choose participants with variations in management levels, work sector, management experiences, educational level, age and gender to create preconditions for a rich dataset. The importance of a dialogue among co-researchers has often been highlighted to achieve dependability, and in this study, all authors were involved in the analysis, and we discussed the themes and subthemes until consensus was reached. We also tried to be aware and open to the fact that the researchers’ pre-understandings could influence the outcome. Also, we considered the importance of deciding which codes and supporting quotes from the text were included in a category. One limitation could be issues regarding translating quotations from Swedish to English, with the risk of something being lost. This limitation was decreased by having all authors review the translations to ensure the meaning of the data was intact following translation. Quotations are described as important quality criterion to achieve credibility (Feldermann & Hiebl, 2020). Another limitation could be that only a few of the middle and senior level managers participated in this study. One explanation could be the low number of managers at higher levels working in these municipalities, and possibly some of them may be concerned of being identified. We think that the findings of this study can likely be transferred to other managers in the municipal organizations in Sweden and other Nordic countries, and our findings are to a large extent supported by earlier research of managers working in the municipal sector outside the rural area. It is also the readers who actually determine the transferability, that is how applicable the findings are to other contexts.

## Conclusion

This study contributes to important knowledge of experiences of working as manager in the municipal sector in rural northern Sweden, and the findings are in line with earlier research describing adverse psychosocial working conditions among managers in Swedish municipal organizations. The managers described being exposed to shrinking resources, conflicting demands, work-related stress and a negative spillover effect on health and family life. This study provides important insights into the managers’ exposed situations in the small municipalities in the rural context, with decreased economy, difficulties in recruiting staff and limited opportunities to finding other work. This study highlights motivation, social support and coping strategies as important elements, as well as the advantages of working in small municipalities, for example, great opportunities to influence within the municipality. The results of this study can contribute to important knowledge in the development of methods aiming to improve managers’ occupational well-being and work performance, and organizational productivity. Future studies could focus on applying adapted workplace support interventions among managers in the municipal sector in rural northern Sweden.

## Data Availability

The data that support the findings of this study are only available on request from the corresponding author.
